# Validity of modified early warning, Glasgow Blatchford, and pre-endoscopic Rockall scores in predicting prognosis of patients presenting to emergency department with upper gastrointestinal bleeding

**DOI:** 10.1186/s13049-015-0194-z

**Published:** 2015-12-30

**Authors:** Seyran Bozkurt, Ataman Köse, Engin Deniz Arslan, Semra Erdoğan, Enver Üçbilek, İbrahim Çevik, Cüneyt Ayrık, Orhan Sezgin

**Affiliations:** Emergency Medicine Department, Mersin University Medical Faculty, Mersin, Turkey; Department of Emergency Medicine, Diskapı Yıldırım Beyazit Training and Research Hospital, Ankara, Turkey; Biostatistics and Medical Informatics Department, Mersin University Medical Faculty, Mersin, Turkey; Gastroenterology Department, Mersin University Medical Faculty, Mersin, Turkey

**Keywords:** Scores, Emergency department, Upper gastrointestinal system bleeding

## Abstract

**Background:**

GBS, MEWS, and PER scoring systems are not commonly used for patients presenting to emergency department with GIS bleeding. This study aimed to determine the value of MEWS, GBS, and PER scores in predicting bleeding at follow-up, endoscopic therapy and blood transfusion need, mortality, and rebleeding within a 1-month period.

**Methods:**

A total of 202 consecutive patients with upper GIS bleeding between July 2013 and November 2014 were prospectively enrolled in the study. The relationship between MEWS, GBS, and PER scores and hospital outcome, bleeding at follow-up, endoscopic therapy, transfusion need, rebleeding, and death were examined.

**Results:**

The study included a total of 202 subjects, with 84 (41.6 %) females and 118 (58.4 %) males. There was a significant correlation between GBS, MEWS, and PER scores and hospital outcomes (*p* <0.004, *p* <0.001, *p* <0.001, respectively). A GBS score greater than 11 succesfully predicted bleeding at follow-up (*p* = 0.0237). GBS score's sensitivity for predicting endoscopic therapy was greater than those of other scoring systems. The discriminatory power of each scoring system was significant for predicting transfusion (*p* <0.0001, *p* = 0.0470, and *p* = 0.0014, respectively). A GBS score greater than 13, a MEWS score greater than 2, and a PER score greater than 3 predicted death. A PER score greater than 3 predicted rebleeding (*p* <0.0001).

**Conclusion:**

The scoring systems in question can be easily calculated in patients presenting to ED with upper GIS bleeding and may be beneficial for risk stratification, determination of transfusion need, prediction of rebleeding, and decisions of hospitalization or discharge.

## Background

Acute upper gastrointestinal system (GIS) bleeding is a common and life-threatening condition [1]. It originates proximal to the ligament of Treitz and forms 85 % of all GIS bleeding episodes [2, 3]. It is associated with an annual incidence of 50–172/100.000, a mortality of 2–15 %, and a rebleeding rate around 10–30 % [4]. The approach to upper GIS bleeding consists of maintenance of hemodynamic stability and determination of the amount and localization of bleeding [5]. The prognosis of GIS bleeding is variable, from mild to life-threatening bleeding [6]. As in all life-threatening conditions in an emergency department (ED), physical examination, diagnostic procedures, and therapeutic efforts should be simultaneously initiated, and patients should be resuscitated and stabilized in upper GIS bleeding [5].

In patients with upper GIS bleeding a triage system for decisions regarding emergency vs delayed endoscopy may shorten hospital stay and cut costs, although this is not always the case. Thus, triage and scoring systems together may categorize patients into low-risk and high-risk groups based on admission criteria prior to endoscopy and may prove more practical [2]. Risk scores based on clinical and endoscopic variables have been developed for patients with acute GIS bleeding [7]. These scoring systems are the widely used Glasgow Blatchford score (GBS) and Rockall scoring systems. Rockall is a scoring system with pre-endoscopic rockall (PER) and endoscopic components. GBS is a scoring system using basic clinical and laboratory variables [2, 8]. Modified early warning score (MEWS) is a simple, physiological, bedside scoring system. It may help recognize critical or potentially critical patients [9, 10].

Risk stratification in upper GIS bleeding has been a topic of research in the last couple of years [11]. Contraversy surrounds risk stratification, role of endoscopic therapy, and indications for medical and surgical treatment. There is, therefore, no consensus on a specific approach to such patients [2]. Early recognition and accurate risk stratification of patients with higher mortality and rebleeding risks may increase the efficiency of patient care while providing guidance for emergency physicians for making final decisions (hospital admission, intensive care unit admission, discharge from ED) [12, 13]. This study aimed to determine the efficiency of GBS, MEWS and PER scores in predicting bleeding at follow-up, need for endoscopic therapy (endoscopic intervention requirement for homeostasis) and blood transfusion, mortality, and rebleeding within 1 month.

## Methods

### Study design

This study was prospectively conducted in patients presenting to the ED of Mersin University Faculty of Medicine with upper GIS bleeding between July 2013 and November 2014. The study was approved by the local ethics committee of Mersin University (Dated 11 July 2013, No: 2013/229). Before starting the study, we carried out a power analysis. A review of medical records yielded 600 patients meeting inclusion criteria who presented to the ED between 1 January 2012 and 31 December 2012 and who were diagnosed with the disorder in question. It is considered that the number of patients presenting to the ED for a time period of 1 year from 15 July 2013 would reach at least that number. Under the light of this information, reaching at least 30 % of the population would suffice to best represent the population [14]. According to the power analysis, we intended to enroll 180 patients with upper GIS bleeding for this study. The criteria of inclusion were patients over the age 18 who presented to ED with melena, hematochesia, and hematemesis and who were confirmed to have upper GIS bleeding by esophagogastroduodonoscopy (EGD) (Fujinon, JAPAN). Patients referred by another institution after initial treatment, those who did not undergo EGD, those whose bleeding was not of upper GIS origin, and those who could not be followed within the first month after discharge were excluded. In addition, pregnant and trauma patients were also excluded. All patients presenting to ED with upper GIS bleeding received an consisting of proton pump inhibitors, fluid resuscitation, and securing airway and breathing. Age, sex, presenting complaint (hematochesia, hematemesis, melena, syncope, active bleeding), rectal examination findings, type and amount of transfusion if any, endoscopy findings, endoscopic and surgical interventions, bleeding at follow-up, hospital outcome (discharge from ED, hospitalization, death), and rebleeding within 1 month after admission were recorded in pre-prepared data forms. A total of 202 consecutive patients with upper GIS bleeding were prospectively enrolled in the study. ED staff and endoscopists not were aware of the study.

GBS, MEWS and PER scores were calculated according to clinical and laboratory data, as described in the original articles [4, 8, 10] (Tables 1 and 2). The correlation of these scores to hospital outcome, bleeding at follow-up, need for endoscopic therapy (endoscopic intervention requirement for homeostasis), transfusion need, rebleeding, and death was examined.

**Table 1:** Glasgow-Blatchford score, pre-endoscopic rockall score, modified early warning score

Glasgow-Blatchford score	
Admission risk marker	Score component value
Blood urea nitrogen (mg/dL)	
≥ 18.2 <22.4 mg/dL	2
≥ 22.4 <28 mg/dL	3
≥ 28 <70 mg/dL	4
≥ 70 mg/dL	6
Hemoglobin for men (g/dL)	
≥ 12 <13 g/dL	1
≥ 10 <12 g/dL	3
<10 g/dL	6
Hemoglobin for women (g/dL)	
≥ 10 <12 g/dL	1
<10 g/dL	6
Systolic blood pressure	
≥ 100 <109 mm Hg	1
≥ 90 <99 mm H	2
<90 mm Hg	3
Other markers	
Pulse ≥100/min	1
Presentation with melena	1
Presentation with syncope	2
Hepatic disease	2
Heart failure	2
Pre-endoscopic rockall score	
Admission risk factor	Score component value
Age, y	
<60	0
60–79	1
≥ 80	2
Shock	
No shock: SBP ≥100, pulse <100	0
Tachycardia: SBP ≥100, pulse ≥100	1
Hypotension: SBP <100	2
Comorbidity	
No major comorbidity	0
Cardiac failure, ischemic heart disease, any major comorbidity	2
Renal failure, liver failure, disseminated malignancy	3

*SBP* systolic blood pressure

**Table 2:** Glasgow-Blatchford score, pre-endoscopic rockall score, modified early warning score

Modified early warning score							
Score	3	2	1	0	1	2	3
SBP mmHg	<70	71–80	81–100	101–199	–	>200	–
Heart rate, bpm	–	<40	41–50	51–100	101–110	111–129	>130
Respiratory rate, rpm	–	<9	–	9–14	15–20	21–29	>30
Temperature, 1C	–	<35	–	35.0–38.4	–	>38.5	–
AVPU	–	–	–	A	V	P	U

AVPU: *A* alert, *V* reacting to voice, *P* reacting to pain, *U* unresponsive

Bleeding at follow-up was defined as recurrent hematemesis, coffee ground vomitus or hemodynamic instability coupled with ongoing melena or hematocrit drop. Rebleeding is defined as hematemesis, coffee ground vomitus, or melena, blood in feces, and admission to hospital for this reason within a 1-month period after discharge from ED or hospital ward. Death was defined as loss of life in ED or hospital ward. Endoscopic therapy was defined as isotonic saline, epinephrine (1 mg, 1/1000, Biopharma, Turkey) or sclerosing agent injection (sclerotherapy) (aethoxysklerol ampul %1, Kreussler pharma, German) electrocoagulation (Erbe, German), clips (Olympus, Japan) and band ligation (Boston scientific, USA) applications. Blood transfusions were used in patients with a hemoglobin level below 10 g/dl and hemodynamic instability despite fluid resuscitation [15].

### Statistical analysis

The statistical analyses were performed with the MedCalc, v15.4. Demo version (MedCalc Software, Ostend, Belgium, http://www.medcalc.be/). Normality of the continuous variables was tested using Shapiro Wilk test. Student *t* test was used to test the differences in mean age by gender. The differences between GBS, MEWS, and PER scores with respect to regular ward or intensive care admission were analyzed with Mann Whitney *U* test while their differences with regard to hospital outcome were tested using Kruskal Wallis test. Paired comparisons of these parameters was performed with Mann Whitney *U* test. Descriptive statistics were presented as mean and standard deviation for normally distributed variables and minimum, maximum, median, and 25–75 % percentiles for non-normally distributed variables. Receiver operator characteristic (ROC) curve analysis were made for GBS, MEWS and PER scores for bleeding, endoscopic treatment, necessary of blood transfusion and recurrent bleeding within a month and the comparision of these scores. Each parameter was classified according to the cut off values that measured. According to the calculated cut-off value was reclassified each parameter. As the descriptive statistics, receiver operating characteristic curve (AUC), sensitivity, specifity, positive predictive value (PPV), negative predictive value (NPV), likelihood ratio (LR) + and LR- values and 95 % confidence intervals (CI) were provided. A p value of less than 0.05 was considered statistically significant.

## Results

### Demographic and clinical data

The annual number of patients presenting to the ED of our hospital was 65,160, of which 0.22 % were the upper GIS bleeding cases. During the study period (July 2013 and November 2014) a total of 97,754 patients were admitted to our ED, of whom 219 suspected with upper GIS bleeding. Two patients refused endoscopy, 7 had non-upper GIS bleeding, 8 were lost to follow-up by 1 month and all of them were excluded from the study.

The study included a total of 202 subjects, with 84 (41.6 %) females and 118 (58.4 %) males. The mean age of the study population was 61.1 ± 17.3 years. The mean age of the female and male subjects were 63.3 ± 18.7 and 59.6 ± 16.2 years, respectively and no differences between the sex had been observed (*p* = 0.127). Variceal hemorrhages constitute 29.2 % of the bleeding events. Hematemesis and melena were the admission symptoms in 47.5 and 45 % of patients, respectively. Rectal examination revealed melena in 61.9 % of these patients. Of the patients with upper GIS bleeding, 52.7 % underwent sclerotherapy, 74.8 % were admitted to hospital, and 12.6 % of the patients had rebleeding within 1 month after discharge (Table 3).

**Table 3:** Demographical and clinical data of the patients that are included in the study

Data		Number	Percent
Gender	Female	84	41.6
	Male	118	58.4
Complaint	Melena	91	45.0
	Hematemesis	96	47.5
	Hematochezia	5	2.5
	Syncope	1	0.5
	Dizziness	2	1.0
	Stomachache	1	0.5
	Active bleeding^a^	6	3.0
RT finding	Melena	125	61.9
	No Melena	57	28.2
	Rectum empty	12	5.9
	Hematochezia	8	4.0
Endoscopic treatment	Sclerotherapy	29	52.7
	Adrenalin injection	2	3.6
	Band ligation	20	36.4
	Other	4	7.3
Bleeding type	Varicous	59	29.2
	Non varicous	143	70.8
Hospital outcome	Discharge	40	19.8
	Hospitalization	151	74.8
	Death	11	5.4
One month later result	Normal	158	82.7
	Rebleeding	24	12.6
	Death	9	4.7
Hospitalization place	Service	112	69.1
	Intensive care unit	50	30.9
Blood transfusion	Applied	146	72.3
	Not applied	56	27.7
Bleeding at follow-up	Bleeding	34	16.8
	Not bleeding	168	83.2

^a^Active bleeding, fresh red gushing bleeding from mouth and rectum

### Scores and patient outcomes

GBS, MEWS, and PER scores were significantly correlated to hospital outcomes (*p* = 0.004, *p* <0.001, and *p* <0.001, respectively). The median GBS score was significantly greater in the hospitalized patients than the discharged ones (*p* <0.001) and in the non-surviving patients than the discharged ones (*p* = 0.001). The non-surviving patients had a significantly greater median MEWS score compared to the discharged and hospitalized ones (*p* = 0.001 and *p* = 0.003, respectively). The median PER score was significantly greater in both hospitalized and non-surviving patients compared to the discharged ones (*p* = 0.002 and *p* <0.001). Furthermore, median PER score of the non-surviving patients was significantly greater than the hospitalized patients (*p* = 0.007) (Table 4).

**Table 4:** Value of the scores according to hospital outcome

		GBS		MEWS		PER	
		Min-Max	Median [25–75 percentile]	Min-Max	Median [25–75 percentile]	Min-Max	Median [25–75 percentile]
Hospitalization place	Service (*n* = 112)	1–18	12 [10–14]	1–8	2 [1–3]	0–7	3 [2–4]
	Intensive care unit (*n* = 50)	6–17	13 [10.75–15.00]	1–9	2.5 [2–4]	0–6	5 [3–5]
	*p*	0.004		0.004		<0.001	
Hospital outcome	Discharge^a^ (*n* = 40)	2–18	9 [5–12]	1–5	2 [1–3]	0–6	2 [0.25–4.00]
	Hospitalization (*n* = 151)	1–18	12^b^ [10–14]	1–8	2 [1–3]	0–7	3^b^ [2–5]
	Death (*n* = 11)	9–17	14^b^ [11–15]	2–9	4^b,c^ [2–5]	3–6	5^b,c^ [4–5]
	*p*	0.004		<0.001		<0.001	

*Abbreviations*: *GBS* Glasgow Blatchford score, *MEWS* modified early warning score, *PER* pre-endoscopic rockall

^a^Discharge containing the patients discharged from the emergancy service. ^b^Shows differance between discharge; ^c^shows differance between hospitalizing

### ROC curve analyses of the scores and the parameters

**Fig. 1:**
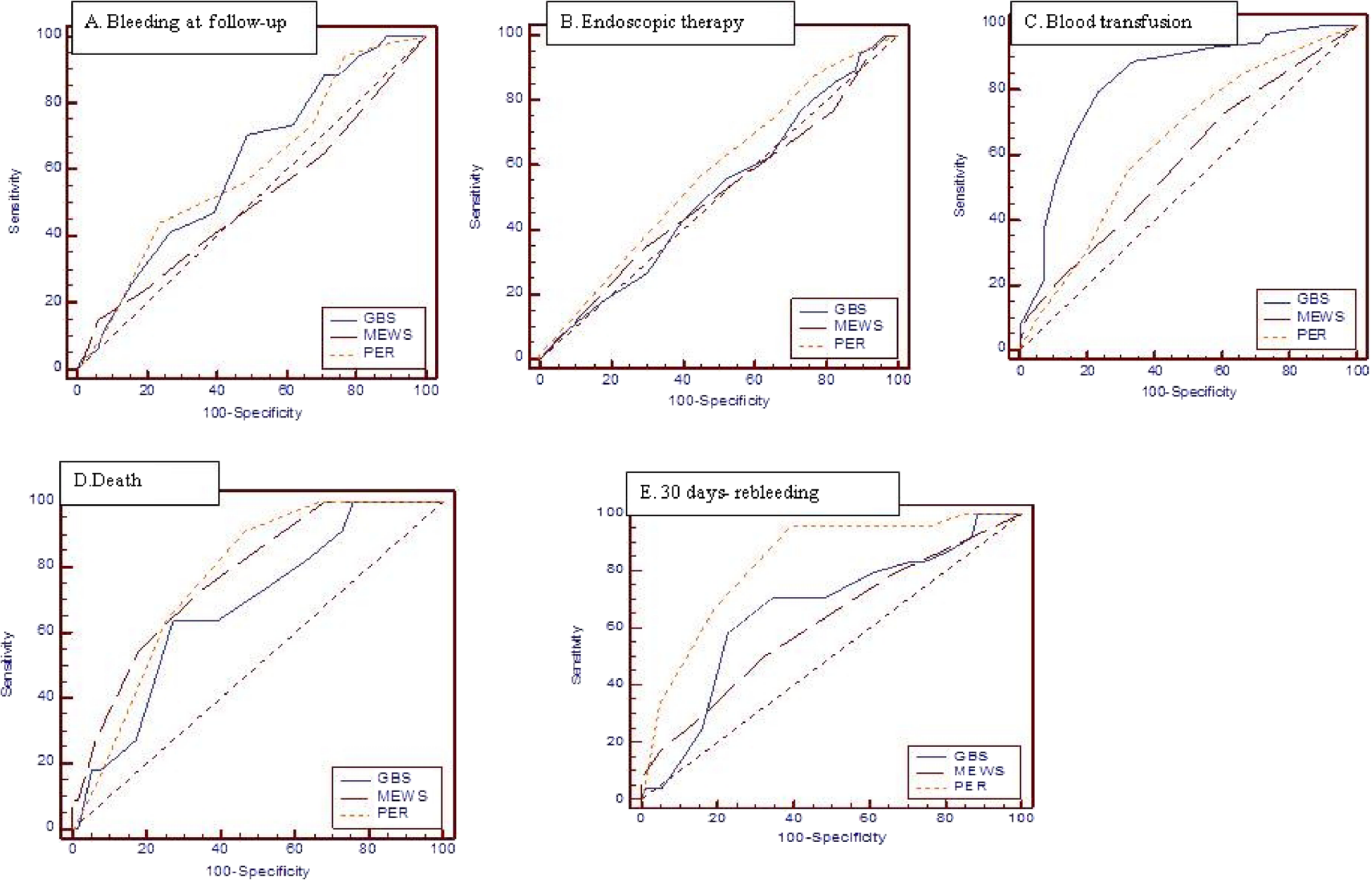
**a**–**e**. The comparison of ROC analysis of GBS, MEWS and PER scores. Bleeding at follow-up (**a**), endoscopic therapy (**b**), blood transfusion (**c**), mortality (**d**), 30 days- rebleeding (**e**), the predicting sensitivity and specificity of scores

ROC curve analyses for continuous variables regarding bleeding at follow-up revealed that only the GBS score's predictive ability was statistically significant, with a GBS score greater than 11 predicted bleeding at follow-up (*p* = 0.0237). MEWS and PER scores greater than 4 predicted bleeding at follow-up, although they did not reach statistical significance (*p* = 1.000 and *p* = 0.055). MEWS had a greater specifity (94.1 %) for bleeding at follow-up than the other two scores (Table 5, Fig. 1a).

**Table 5:** The ROC analysis results of the continuous variables

		Cut off	AUC (*p* value)	Sensitivity [95 % CI]	Specifity [95 % CI]	PPV [95 % CI]	NPV [95 % CI]	LR+ [95 % CI]	LR- [95 % CI]
Bleeding at follow-up	GBS	>11	0.613 **(0.0237)**	70.59 [52.5–84.9]	51.19 [43.4–59.0]	22.64 [15.1–31.8]	89.58 [81.7–94.9]	1.45 [1.1–1.9]	0.57 [0.3–1.0]
	MEWS	>4	0.500 (1.000)	14.71 [5.0–31.1]	94.05 [89.3–97.1]	33.33 [11.8–61.6]	84.49 [78.5–89.4]	2.47 [0.9–6.8]	0.91 [0.8–1.0]
	PER	>4	0.600 (0.0550)	44.12 [27.2–61.1]	76.19 [69.0–82.4]	27.27 [16.1–41.0]	87.07 [80.6–92.0]	1.85 [1.2–3.0]	0.73 [0.5–1.0]
Endoscopic therapy	GBS	>5	0.514 (0.7501)	94.64 [85.1–98.9]	10.49 [6–16.7]	29.28 [22.8–36.5]	83.33 [58.6–96.4]	1.06 [1–1.1]	0.51 [0.2–1.7]
	MEWS	>1	0.508 (0.8623)	33.93 [21.8–47.8]	71.33 [63.2–78.6]	31.67 [20.3–45.0]	73.38 [65.2–80.5]	1.18 [0.8–1.9]	0.93 [0.7–1.1]
	PER	>3	0.577 (0.0769)	57.14 [43.2–70.3]	54.55 [46.0–62.9]	32.99 [23.8–43.3]	76.47 [67.0–84.3]	1.26 [0.9–1.7]	0.79 [0.6–1.1]
Need for blood transfusion	GBS	>10	0.829 **(<0.0001)**	79.45 [72.0–85.7]	76.79 [63.6–87.0]	89.92 [83.4–94.5]	58.90 [46.8–70.39	3.42 [2.1–5.6]	0.27 [0.2–0.4]
	MEWS	>1	0.584 **(0.0470)**	73.29 [65.3–80.3]	39.29 [26.5–53.2]	75.89 [68.0–82.7]	36.07 [24.2–49.4]	1.21 [1.0–1.5]	0.68 [0.4–1.0]
	PER	>3	0.642 **(0.0014)**	55.48 [47.0–63.7]	67.86 [54.0–79.7]	81.82 [72.8–88.9]	36.89 [27.6–47.0]	1.73 [1.1–2.6]	0.66 [0.5–0.8]
Death	GBS	>13	0.679 **(0.0222)**	63.64 [30.8–89.1]	72.77 [65.9–79.0]	11.86 [4.9–22.9]	97.20 [93.0–99.2]	2.34 [1.4–3.9]	0.50 [0.2–1.1]
	MEWS	>2	0.772 **(<0.0001)**	72.73 [39.0–94.0]	65.45 [58.2–72.2]	10.81 [4.8–20.2]	97.66 [93.3–99.5]	2.10 [1.4–3.2]	0.42 [0.2–1.1]
	PER	>3	0.767 **(<0.0001)**	90.91 [58.7–99.8]	53.40 [46.1–60.6]	10.10 [5.0–17.8]	99.03 [94.7–100.0]	1.95 [1.5–2.5]	0.17 [0.03–1.1]
Rebleeding	GBS	>12	0.663 **(0.0073)**	70.83 [48.9–87.4]	65.27 [57.5–72.5]	22.67 [13.8–33.8]	93.97 [88.0–97.5]	2.04 [1.5–2.8]	0.45 [0.2–0.8]
	MEWS	>2	0.617 (0.0638)	50.0 [29.1–70.9]	67.66 [60.0–74.7]	18.18 [9.8–29.6]	90.40 [83.8–94.9]	1.55 [1.0–2.4]	0.74 [0.5–1.1]
	PER	>3	0.832 **(<0.0001)**	95.83 [78.9–99.9]	60.48 [52.6–67.9]	25.84 [17.1–36.2]	99.02 [94.7–100.0]	2.42 [2.0–3.0]	0.07 [0.01–0.5]

*the “Bold numbers” p<0.05. Abbreviations*: *GBS* Glasgow Blatchford score, *MEWS* Modified early warning score, *PER* pre-endoscopic rockall, *ROC* receiver operator characteristic, *AUC* receiver operating characteristic curve, *PPV* positive predictive value, *NPV* negative predictive value, *LR* likelihood ratio, *CI* confidence intervals

ROC curve analyses in patients that underwent endoscopic therapy showed that patients with a GBS score greater 5, a MEWS score greater than 1, and a PER score greater than 3 needed an endoscopic examination. GBS, MEWS and PER scores had very low discriminative abilities for prediction of need of endoscopic therapy (AUC: 0.51, 0.51 and 0.58, respectively) (Table 5, Fig. 1b).

Analysis of ROC curves of the continuous variables in transfused patients showed that all three scoring systems were significantly able to predict transfusion (*p* <0.0001, *p* = 0.0470, and *p* = 0.0014, respectively). According to the analysis, a GBS score greater than 10, a MEWS score greater than 1, and a PER score greater than 3 predicted transfusion need. The values of GBS were higher than those of the other two scoring systems. GBS was significantly more effective than the other two scoring systems in predicting transfusion need (p <0.0001 vs each of the other two systems) (Table 5, Fig. 1c).

In patients who died after upper GIS bleeding the ROC curve analysis of continuous variables demonstrated that the three scores were significantly able to predict death (*p* = 0.0222, *p* <0.0001, and *p* <0.0001). A GBS score greater than 13, a MEWS score greater than 2, and a PER score greater than 3 predicted death. PER score had a greater sensitivity (90.9 %) and NPV (99 %) compared to other two scores (Table 5, Fig. 1d).

ROC curve analysis demonstrated that a PER score greater than 3 significantly predicted rebleeding for discharged patients within 1 month and it was statistically significant (*p* <0.0001). PER score had a sensitivity of 95.8 %, specifity of 60.5 %, and NPV of 99.2 %. A cut-off level of 2 for MEWS and 12 for GBS predicted rebleeding, although their discriminatory ability was not statistically significant (*p* = 0.0638 and *p* = 0.073, respectively). PER score had a greater NPV (99.2 %) compared to other two scores. PER score predicted rebleeding within 1 month significantly better than GBS and MEWS scores (PER and GBS *p* = 0.0055 and PER and MEWS *p* <0.0001) (Table 5, Fig. 1e).

## Discussion

Many risk stratification tools are used in clinical practice, especially for critically-ill patients. Emergency physicians need some tools for making decisions between a safe early discharge with outpatient follow-up and endoscopic examination and follow-up in ED. In patients admitted to ED with the complaint of GIS bleeding, it is not clear which scoring system must be used or what the efficacy of these systems are. We investigated the ability of GBS, MEWS, and PER scores for predicting bleeding at follow-up, need for endoscopic therapy, transfusion need, death, and rebleeding. Our study demonstrated that GBS and PER scores were significantly lower in discharged patients compared to admitted patients. However, MEWS scores of the discharged and admitted patients were similar. This can be explained by a slight increase in pulse and respiratory rates of patients at the early stages of bleeding. A recent study suggested that GBS accurately predicted outcomes including need for intensive care [16]. Hence, scoring systems can provide valuable information concerning the urgency of endoscopy and level of patient care (i.e., whether patient care and follow-up should be made in regular ward or intensive care unit). Our study demonstrated significantly different efficacy in determining ward or intensive care admission. In non-surviving patients MEWS and PER scores were significantly greater than admitted patients whereas no such difference existed for GBS. Subbe et al. reported that death and intensive care unit admission were more frequent in cases with a MEWS score greater than 5 compared to those with a MEWS score less than 5 [17]. In our study MEWS score was significantly greater in non-surviving patients compared to other patients. Another result of our study was that hospitalized and non-surviving patients had no difference with respect to GBS score. GBS is not developed for predicting mortality [18]. This may be explained by the absence of prominent blood urea elevation in early stages of upper GIS bleeding, or lack of hemoglobin drop due to hemoconcentration in patients with blood loss. In study with GIS bleeding, Conflicting results have been reported regarding the role of age in mortality [6, 19, 20]. Although we did not explored the effect of age on mortality, the difference between the median PER scores suggests that age may be a factor in mortality.

Most GIS bleeds improve without blood transfusions, endoscopic therapy, or surgical intervention [20]. Its has been shown by many studies that GBS performs better than Rockall score in predicting adverse events including death, blood transfusion, endoscopic therapy, and surgery in patients with upper GIS bleeding [12, 18, 21]. Studies exploring the value of GBS and PER scores in predicting need for endoscopic therapy, transfusion need, rebleeding, and mortality in upper GIS bleeding have been usually performed on the basis of low risk (score = 0) and high risk (score > 0) [8, 22–24]. One of the striking results of our study was that none of the three scoring systems revealed zero points. This indicates that patients with upper GIS bleeding admitted to tertiary centers like ours are more risky and require more careful management. GBS's sensitivity and specifity for prediction of rebleeding has been reported as 100 and 25 % while they have been reported as 90.2 and 38 % for PER [23]. GBS reportedly predicted the need for endoscopic therapy better than PER [24]. It has been reported that GBS and PER scores had a high sensitivity (100 and 95 %) but a low specifity (4 %, 9 %) [7]. Dicu et al. found no difference between GBS and PER scores for predicting bleeding at follow-up [25]. Our study reveales that GBS alone significantly predicted bleeding at follow-up for scores greater than 11. However, in line with the study of Dicu, ROC curves for bleeding at follow-up produced no difference between GBS and PER scores. For bleeding at follow-up, GBS had a sensitivity of 70.6 %, specifity of 51.2 %, a PPV of 22.6 %, and a NPV of 89.6 %.

GBS can reliably predict need for endoscopic therapy, and need for clinical and surgical intervention [18, 21, 25]. Two studies from the United Kingdom reported that the ROC curve for determining need for clinical intervention remained below 0.92 [6] and 0.90 [26] in about 16 % of low-risk patients. A Japanese study similarly reported an area under curve of 0.63 [27]. We determined that GBS had a greater sensitivity than other scores for predicting the need for endoscopic therapy. In contrast to former studies, however, areas under curve of GBS and PER scores were similar. GBS, MEWS, and PER scores had a sensitivity of 94.6, 33.9, and 57.2 %, respectively; a specifity of 10.5, 71.3, and 57.1 %, respectively; a PPV of 29.3, 31.7, and 33 %, respectively; and a NPV of 83.3, 73.8, and 76.5 %, respectively.

Studies exploring the ability of the scores for predicting transfusion need revealed that GBS had a better predictive ability than PER [18, 21, 25]. Our study determined that GBS performed better than both MEWS and PER for prediction of transfusion need. The sensitivity (79.5 %), specifity (76.8 %), PPV (76.8 %), and NPV (58.9 %) of GBS were greater than those of the other two scores. GBS is a scoring system that considers hemoglobin level, systolic blood pressure and pulse rate of the patients. For that reason it is assumed to better predict the need of transfusion.

Stanley and colleagues reported that GBS and PER scores may predict death with similar accuracy [21]. Subbe et al. found a higher death and intensive care unit admission rate in patients with a MEWS score greater than 5 compared to those with a score less than 5 [17]. Our study demonstrated that MEWS scores greater than a cutoff value of 2 can predict death. In addition, all three scores could pedict death with no significant differences between their areas under curve. PER score had a greater sensitivity (90.9 %) and NPV (99 %) than other scores for mortality prediction. GBS is superior to PER score in prediction of low-risk patients [20]. Our study also indicated that rebleeding might occur within a 1-month period with a PER score greater than 3 and it was statistically significant. The sensitivity of PER for rebleeding was 95.8 %, specifity 60.5 %, and NPV 99.2 %. It was determined that rebleeding may be predicted, albeit statistically non-signifiacantly, by a MEWS cutoff level of 2 and the cutoff levels determined for GBS.

### Limitations

There are some limitations of the present study. First, it was a single-center, low-volume study. Nevertheless, most critical patients are referred to our department since our hospital is a university hospital providing tertiary care service. However, multi-center, high-volume studies may still be conducted. Second, blood transfusion need is not a parameter for determination of endoscopy need. The indication for endoscopy and clinical condition were taken into account only. Decisions about endoscopic examination were made by gastroenterologists and with appropriate criteria. Inter-individual differences may still exist, however.

## Conclusion

GBS was found to be better with respect to prediction of transfusion need and PER score was found to be better in prediction of re-bleeding during the 1-month follow-up period. In conclusion it was observed that none of these current scoring systems are solely superior than the other and in fact they are complimentary to each other. It was seen that there is no single efficient scoring system in clinical practice and they all have some superior and inferior properties.

## Abbreviations

AUC: receiver operating characteristic curve
CI: confidence intervals
ED: emergency department
EGD: esophagogastroduodonoscopy
GBS: Glasgow Blatchford score
GIS: gastrointestinal system
LR: likelihood ratio
MEWS: modified early warning score
NPV: negative predictive value
PER: pre-endoscopic rockall
PPV: positive predictive value
ROC: receiver operator characteristic
SBP: systolic blood pressure
